# Connected or chained by social media? Child and adolescent mental health in a digital era

**DOI:** 10.1371/journal.pmed.1005099

**Published:** 2026-05-11

**Authors:** Silja Kosola

**Affiliations:** Pediatric Research Center, Helsinki University Hospital and University of Helsinki, Helsinki, Finland

## Abstract

In this Perspective, Silja Kosola outlines how social media disproportionately harms child and adolescent mental health, and argues that while recent policy changes aimed at protecting youth from social media are welcome, stricter age limits and greater accountability of social media companies are needed.

## Teaser

Social media has evolved from connection to compulsion, disproportionately harming children and adolescents. Addictive designs together with developmental vulnerability fuel mental health risks and highlight the urgent need for stricter age limits and stronger protections.

The advent of social media meant faster connections to friends, even across oceans. A decade ago, these connections began to morph into chains; an increasing proportion of young people now report being online even when they don’t want to be [1]. Although the social media experiences of children and adolescents may be heterogenous and dependent on gender or the platform used, increasing evidence points to more harm than benefit. The younger a child is when they start using social media, the more likely they are to have mental health issues a few years later [2]. The more adolescents use social media, the more they suffer from anxiety, and the more frequently they report body dissatisfaction, problems concentrating, risky behavior, and ironically, loneliness [2–6].

Since its genesis, social media has undergone a rapid evolution. The earliest changes utilized the psychology of social rewards: likes and followers indicated social acceptance [7]. During the past decade, algorithms have been further developed to incorporate addictive features familiar from casinos, including fast-paced interactions, sudden surprises, and personalization of content. These features beguile users of all ages, but the effect is greatest on children and adolescents due to their developmental stage.

In the adolescent brain, the first area to mature is the limbic system which is responsible for emotions, sensation-seeking, and social rewards [8]. The last brain areas to mature are in the prefrontal cortex and involve impulse control, delayed gratification, and identity. Due to this imbalance between sensation-seeking and impulse control, adolescents are especially susceptible to the dopamine-inducing fast pace and surprising nature of short videos, which provide excitement and thus increase the likelihood of addictive behavior and attention deficit. Insecure about their identity, adolescents have often sought idols, which makes them sensitive to the endless upward comparisons in social media and may explain subsequent body dissatisfaction. Further, the effortlessness of online communication is linked to social anxiety in real life, while personalized content may lead to echo chambers, distortion of values, and decreased trust. In addition, when adolescents spend 5–6 hours daily on their smartphones [3], this time is inevitably away from elements vital to mental health: sleep and physical activity.

**Fig 1:**
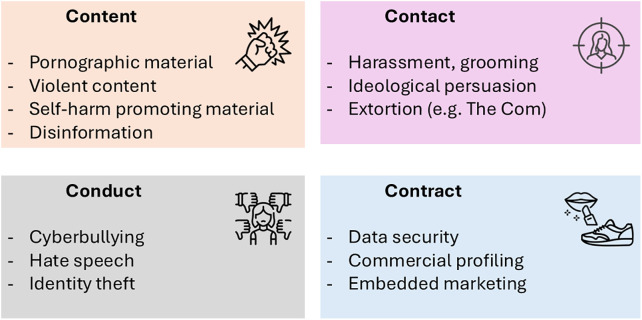
The four C’s of social media harms. The four C’s described by Livingstone and Stoilova [9] depict the potential harms of social media. All social media users are susceptible to these harms but due to adolescent brain development and limited life experience, children and adolescents are at greatest risk.

In addition to the counterproductive algorithms, the potential harms of social media for children have been divided under four C’s: content, contact, conduct, and contract (see Fig 1) [9]. In contrast to movies and games, which experts assess according to the Pan European Game Information (PEGI) age labels, no content review is available for social media. As to contact, lonely children are at greatest risk of online exploitation because, seeking acceptance, they reveal secrets that are used for extortion [10]. Conduct issues mirror how screens distance people, and bullying prevails on all popular social media and gaming sites. Contract refers to the exploitation of child and adolescent user data and inappropriate exposure to commercial content.

The logical solution to the problems caused by social media would be for social media companies to act responsibly and change the course of development for the platforms. Unfortunately, existing efforts to protect children from harm are insufficient. Meta has left fact-checking to individual users, while former employees attest that companies are aware of the problems they are causing [11]. In a landmark case in California, TikTok and Snapchat chose to settle outside of court, and Meta and Google were found liable because of the addictive nature of infinite scrolls, autoplay, and notifications [12]. As a first step to protect children, the age limit of social media should be raised to 16 years, in line with PEGI labels and general child protection laws.

Australia was the first country to enforce a legal age limit of 16 years for social media in December 2025. The first news was impressive: nearly 5 million accounts of under-aged users were disabled. Thereafter, however, problems emerged because social media companies in charge of age verification utilize various weak methods, including face recognition and user profiling, which make restrictions easy to circumvent [13]. Some adolescents also moved to other, unrestricted platforms.

Most problems with the new age limits could be easily avoided. Instead of leaving the responsibility of age verification to parents who already struggle with the existing age limit of 13 years or to companies that have shown little interest in child and adolescent well-being, use of zero-knowledge proof systems already implemented in several European countries could be required. Presenting cryptographic proof of age would provide both data security and reliable age verification [13]. This would ensure stronger enforcement than with movies and games with attached PEGI labels. Instead of laws listing particular platforms, it may be more practical to list addictive algorithm features and types of functions that make platforms unsuitable for users under 16. Consequently, the laws would be more difficult to circumvent, and legal text would need fewer updates. Furthermore, engaging with youth and families in policy design could increase acceptability. Globally, many countries are following Australia’s example. In Europe, Denmark and Norway were the first countries to start planning higher age limits for social media. In the proposed Danish model, the age limit would be 15, but parents could grant permission for younger children to establish social media accounts. Despite the good intention of parental empowerment, this creates a new potential pitfall; leaving parents to struggle with children and adolescents who argue that “everyone else” is allowed to use social media could undermine the age limit or lead to increased fear of missing out and psychosocial distress. Young people themselves tend to accept restrictions better if the same rules apply to everyone [14]. Legal age limits also protect children in families where parents or guardians are unable to set limits for their offspring, thus reducing social inequity. Several members of the European Parliament have called for an EU-wide minimum age, which would be even more powerful than laws of individual countries and strengthen cross-jurisdictional coordination. Building a European legal framework could affect general attitudes in society as seen previously with seatbelts and other traffic safety measures.

It is the responsibility of adults to keep children safe both online and in the real world and to teach them the skills they need to navigate both worlds on their own. Still, children and adolescents should only engage in activities suitable to their developmental stage. Social media platforms were never intended for children, and safer technology exists for seeking information and for digital connections with relatives and peers. The proposed age limits are only part of the solution. In addition, schools still need to teach children media literacy and online safety. Well-child clinics and other health and social services should provide families with support and guidance for health-promoting digital habits. Finally, governments should monitor compliance of social media companies and remain vigilant about future developments, especially regarding the use of artificial intelligence and its effects on children, adolescents, and society at large. After adjustments, the feeling of connection could be maintained without the current constricting chains, potentially leading to improved mental health.

## Abbreviation:

PEGI: Pan European Game Information
